# Comparative study of analgesia nociception index (ANI) vs. standard pharmacokinetic pattern for guiding intraoperative fentanyl administration among mastectomy patients

**DOI:** 10.1186/s12871-021-01272-2

**Published:** 2021-02-13

**Authors:** Sirirat Tribuddharat, Thepakorn Sathitkarnmanee, Pornlada Sukhong, Maneerat Thananun, Parinda Promkhote, Duangthida Nonlhaopol

**Affiliations:** Department of Anesthesiology, Faculty of Medicine, Khon Kaen University, 123 Mitrapap road, Ampur Muang, Khon Kaen, 40002 Thailand

**Keywords:** Analgesia nociception index, ANI, Monitor, General anesthesia, Intraoperative fentanyl, Pain score, Pharmacokinetic pattern

## Abstract

**Background:**

The Analgesia Nociception Index (ANI) has been suggested as a non-invasive guide for analgesia. Our objective was to compare the efficacy of ANI vs. standard pharmacokinetic pattern for guiding intraoperative fentanyl administration.

**Methods:**

This was a prospective, randomized, controlled study of adult female patients undergoing elective mastectomy under general anesthesia. The patients were randomized to the ANI-guided group receiving a loading dose of 75 μg of fentanyl followed by 25 μg when the ANI score was under 50. The Control group received the same loading dose followed by 25 μg every 30 min with additional doses when there were signs of inadequate analgesia (viz., tachycardia or hypertension).

**Results:**

Sixty patients—30 in each group—were recruited. Although the actual mean ANI score was higher in the ANI-guided than in the Control group (mean difference 2.2; 95% CI: 0.3 to 4.0, *P* = 0.022), there was no difference in the primary outcome—i.e., intraoperative fentanyl consumption (mean difference − 4.2 μg; 95% CI: − 24.7 to 16.4, *P* = 0.686 and − 0.14 μg·kg^− 1^·h^− 1^; 95% CI: − 0.31 to 0.03, *P* = 0.105). No difference between groups was shown for either intraoperative blood pressure and heart rate, or for postoperative outcomes (i.e., pain scores, morphine consumption, or sedation scores) in the postanesthesia care unit.

**Conclusions:**

Intraoperative fentanyl administration guided by ANI was equivalent to that guided by a modified pharmacologic pattern. In a surgical model of mastectomy, the ANI-guided intraoperative administration of fentanyl had no impact on clinical outcomes.

**Trial registration:**

The study was registered with ClinicalTrials.gov (NCT03716453) on 21/10/2018.

## Introduction

Drugs used in balanced anesthesia comprise 3 main groups: hypnotics, opioids, and muscle relaxants. To maintain proper depth of anesthesia, all these drugs must be maintained at their therapeutic levels; thus, reliable monitors are needed. The hypnotic state can be monitored using the bispectral index (BIS) for intravenous drugs [1], or the minimum alveolar concentration (MAC) for volatile anesthetics [2]. Muscle relaxation can be monitored with train-of-four ratio using a peripheral nerve stimulator [3]. By contrast, opioid drugs lack a reliable monitor. The administration of opioids depends on the pharmacokinetic pattern of the drug [4] as well as clinical signs (e.g. tears, pupil dilation, sweat, tachycardia, or hypertension). The low specificity of these signs may lead to under- or over-dosage of opioid which may result in intraoperative movement, postoperative pain, nausea and vomiting (PONV), and/or respiratory depression [5].

The Analgesia Nociception Index (ANI; MetroDoloris Medical Systems, Lille, France)—developed from electrocardiogram (ECG) tracing—has been suggested as a noninvasive guide for analgesia. ANI is derived from heart rate variability (HRV) analysis—which provides a measure of the effect of respiratory sinus arrhythmia (RSA) on heart rate through the parasympathetic reflex loop. A painful stimulus induces a decrease in parasympathetic tone leading to a decrease in ANI scores. ANI scores vary between 100, which indicates a state of maximum parasympathetic tone and a low nociceptive level, and 0, which represents the minimum parasympathetic tone and a high nociceptive level. A value ≥50 indicates adequate analgesia [6, 7]. Several studies have shown that ANI could be used to predict immediate postoperative pain [8], guide intraoperative opioid administration [5, 9, 10], and predict the need for analgesia during the early postoperative period [11]. Even though the ideal method for optimizing opioid administration is to follow the pharmacokinetic pattern in order to maintain a serum concentration above the therapeutic level, this method is not practicable. Our research question sought to determine whether the ANI score might be used as a proxy for the pharmacokinetic pattern so as to optimize intraoperative opioid administration. We designed a study comparing intraoperative fentanyl requirement guided by ANI vs. standard pharmacokinetic pattern. The aim of the current study was to compare the efficacy of ANI with standard pharmacokinetic pattern to guide intraoperative fentanyl administration.

## Methods

The current study was approved by the Khon Kaen University Ethics Committee for Human Research (HE611339) and was registered with ClinicalTrials.gov (NCT03716453) on 21/10/2018. All patients gave written informed consent before being recruited. The study was conducted in accordance with the Declaration of Helsinki and the ICH GCP at Srinagarind Hospital, Khon Kaen University between October 2018 and November 2019. The results were reported according to the CONSORT (Consolidated Standards of Reporting Trials) guidelines.

This was a prospective, double-blind (patient and assessor masked), randomized, controlled study. The sample size of 30 per group was based on intraoperative fentanyl consumption (150.83 ± 26.6 μg) after mastectomy in a previous study [12]. The *α*-value was 0.05, the power 0.80, the expected mean difference of intraoperative fentanyl consumption 25 μg, and the expected dropout 25%. Randomization was achieved using block of 4 at a 1:1 ratio using a computer-generated program with lists kept in sealed opaque envelopes. The inclusion criteria were female patients between 18 and 75 years of age undergoing elective mastectomy under general anesthesia. Patients had an ASA physical status between I-II, and a BMI between 18.5 and 35 kg·m^− 2^. The exclusion criteria were patients (a) with implanted pacemaker, cardiac arrhythmia, autonomic nervous system disorder, or chronic pain, (b) taking opioids, beta- or calcium channel blockers, anti-arrhythmic drugs, preoperative nonsteroidal anti-inflammatory drugs (NSAID), or regional block; (c) having previously undergone a mastectomy; or, (d) being pregnant.

All patients were randomized into two groups: ANI-guided or Control. The monitors comprised electrocardiogram, pulse oximeter, non-invasive blood pressure, capnography, temperature, MAC, and ANI. The ANI presents 2 ANI values on the display, i.e., the instantaneous fluctuating index and the medium trend index with a 4-min average reflecting patient tendency. The medium trend index is recommended for patient monitoring. The ANI in the Control group was covered with an opaque cloth so that the attending anesthesiologist did not see the information. At the end of anesthesia, the data from the ANI (medium trend indices) were downloaded for analysis. The patients and outcome assessors were blinded.

The primary outcome was the amount of fentanyl intraoperatively administered. The secondary outcomes were intraoperative ANI scores, blood pressure and heart rate, pain scores in the postanesthesia care unit (PACU) at 0, 15, 30, 45, 60 min, sedation score, and total morphine consumption in PACU.

All patients received fentanyl 75 μg as premedication. Propofol 2 mg·kg^− 1^ was given as the induction agent. Endotracheal intubation was facilitated using cisatracurium 0.15 mg·kg^− 1^. End tidal desflurane was adjusted between 3 and 4% (according to age-adapted MAC) in a N_2_O to O_2_ ratio of 0.5:0.5 L·min^− 1^ to achieve 1 MAC on the monitor so as to maintain depth of anesthesia. Cisatracurium was used for myorelaxation.

During maintenance, the ANI-guided group received fentanyl to maintain an ANI between 50 and 70. When the average ANI score fell below 50 for 30 s, 25 μg of fentanyl was injected. If the ANI score persisted below 50 after 5 min, another dose of 25 μg of fentanyl was repeated until ANI score was ≥50. The Control group received a standardized protocol modified from the reported pharmacokinetic pattern [4] of 25 μg of fentanyl every 30 min as a maintenance dose with additional doses given when there were signs of inadequate analgesia (viz, tachycardia or hypertension), at the discretion of the attending anesthesiologist. Dexamethasone 8 mg and ondansetron 8 mg were given to prevent postoperative nausea/vomiting (PONV). At the end of surgery, neostigmine 2.5 mg plus atropine 1.2 mg were administered as reversal agents. After full recovery, the patients were extubated and transferred to PACU. At the PACU, the patients were evaluated for postoperative pain using a numeric rating scale (NRS) at 0, 15, 30, 45, and 60 min. If the NRS was > 4, then 2 mg of morphine were intravenously administered. This was repeated every 5 min—up to 3 doses. Total doses of morphine in the PACU were recorded. Side effects, i.e., sedation score (0 = wide awake; 1 = easy to rouse; 2 = easy to rouse but unable to remain awake; 3 = difficult to rouse), PONV, and respiratory depression (respiratory rate ≤ 8/min) were also recorded. All data were analyzed with an intention-to-treat approach.

### Statistical analyses

Continuous data were tested for Gaussian distribution using the Shapiro-Wilk test. Data with a normal distribution were presented as means ± standard deviation (SD) while non-Gaussian data were presented as medians and interquartile range. Categorical data were presented as numbers (%). Differences between both groups were analyzed using the unpaired Student’s t-test, Mann-Whitney U test, χ^2^ test, linear mixed model, two-way ANOVA, or Friedman test as appropriate. *P* < 0.05 was considered statistically significant. Statistical analyses were performed using SPSS 16.0 for Windows (SPSS, Chicago, IL, USA).

## Results

**Fig. 1:**
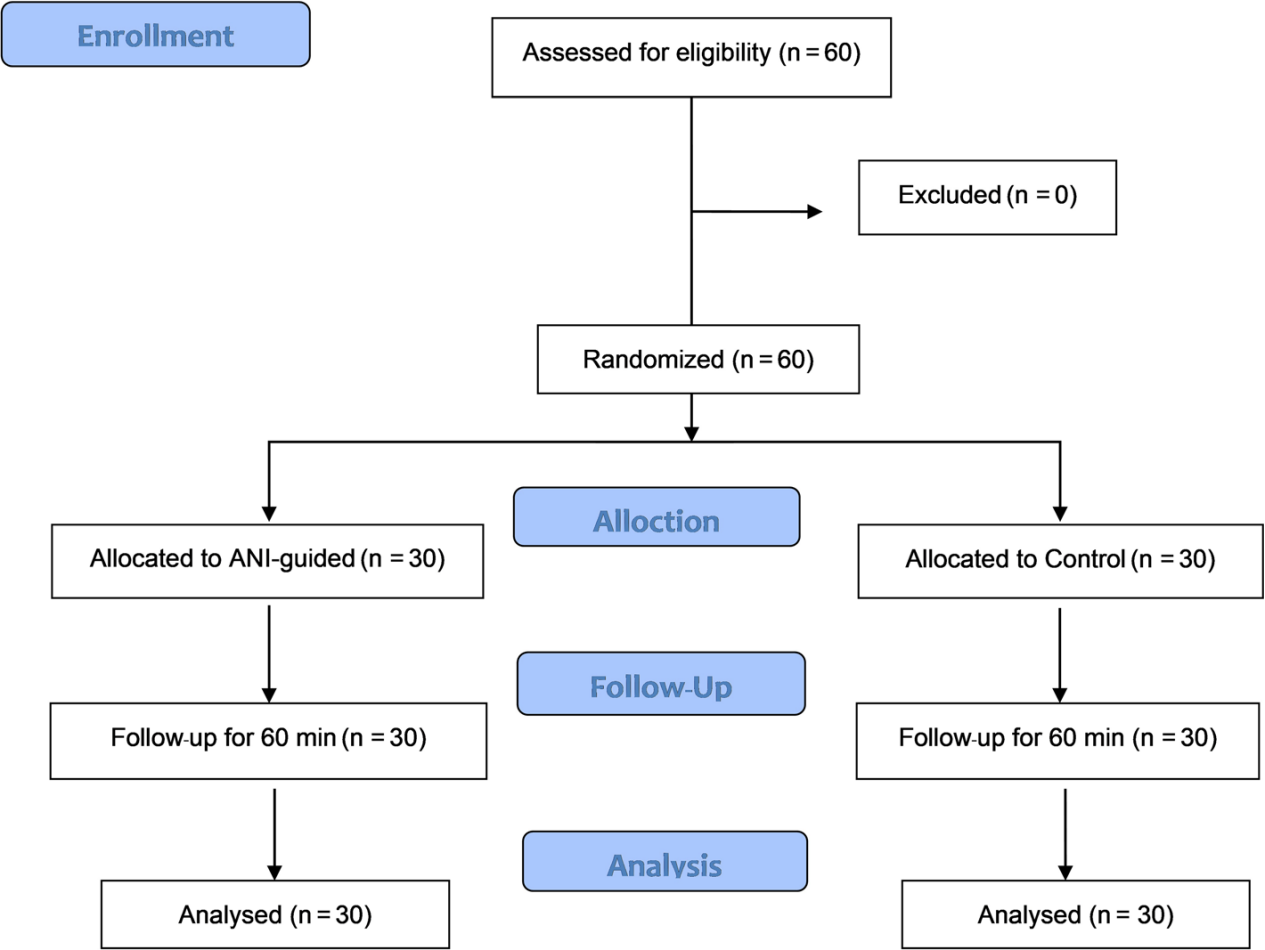
CONSORT diagram of the study. ANI, Analgesia Nociception Index

Sixty patients with 30 in each group were recruited and analyzed (Fig. 1). There were no dropouts. The characteristics and duration of anesthesia of both groups were similar (Table 1).

**Table 1:** Characteristics and clinical data of patients

Variable	ANI-guided group (*n* = 30)	Control group (*n* = 30)	*P-*value
Female	30 (100)	30 (100)	n/a
Age (y)	52.8 ± 12.2	54.8 ± 9.8	0.486
Weight (kg)	58.4 ± 10.1	60.0 ± 10.2	0.551
Height (cm)	155.1 ± 6.1	157.7 ± 5.7	0.099
BMI (kg·m^−2^)	24.2 ± 3.9	24.0 ± 3.6	0.877
ASA status I/II	9 (30)/21 (70)	11 (36.7)/19 (63.3)	0.300
Operation			1.000
Modified radical mastectomy	16 (53.3)	17 (56.7)	
Simple mastectomy	14 (46.7)	13 (43.3)	
Duration of anesthesia (min)	170.8 ± 43.8	152.0 ± 52.7	0.138

Data are expressed as mean ± SD or number (%)

*ANI* Analgesia Nociception Index, *BMI* Body mass index, *ASA* American Society of Anesthesiologists

**Fig. 2:**
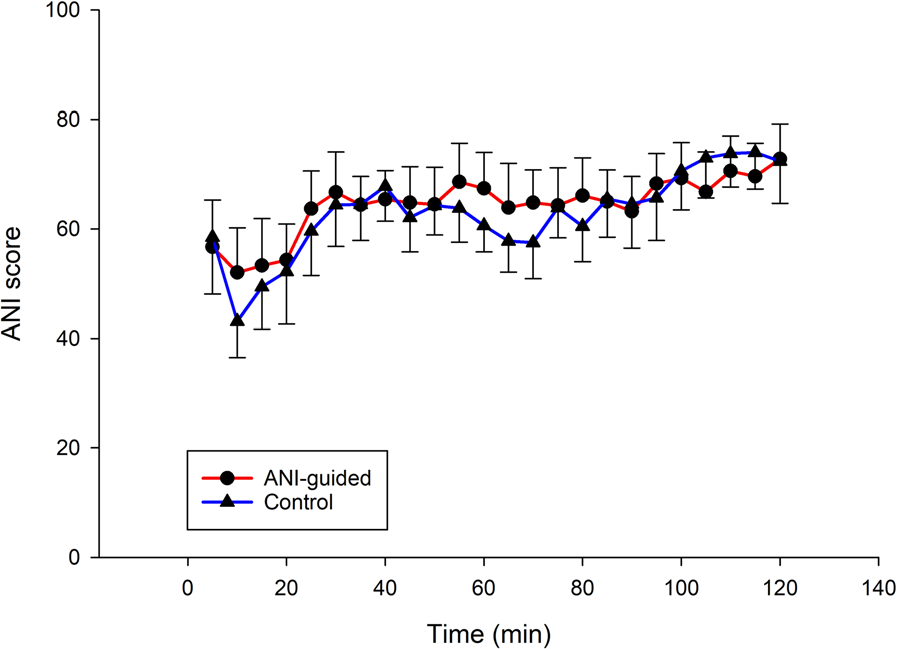
Intraoperative ANI score of both groups. Error bar represents 95% CI. ANI, Analgesia Nociception Index; Time 0, the beginning of surgery

Intraoperative fentanyl consumption both in total dose (μg) and average dose (μg·kg^− 1^·h^− 1^) were similar between groups. The mean ANI scores of the ANI-guided group were higher than the Control group (Table 2). The actual intraoperative ANI scores—measured every 5 min for both groups—are shown in Fig. 2. The type of surgery had no effect on the primary outcome (*P* = 0.613, adjusted analysis).

**Table 2:** Intraoperative fentanyl consumption and ANI score

Variable	ANI-guided group (*n* = 30)	Control group (*n* = 30)	Mean difference (95% CI)	*P-*value
Total intraoperative fentanyl consumption				
Total dose (μg)	158.3 ± 38.5	162.5 ± 40.9	−4.2 (−24.7 to 16.4)	0.686^†^
Average dose (μg·kg^−1^·h^−1^)	1.01 ± 0.33	1.16 ± 0.34	−0.14 (− 0.31 to 0.03)	0.105^†^
Mean ANI score	65.2 ± 17.7	63.0 ± 18.2	2.2 (0.3 to 4.0)	0.022^†^

Data are expressed as mean ± SD; ^†^Unpaired Student’s t-test

*ANI* Analgesia Nociception Index

**Fig. 3:**
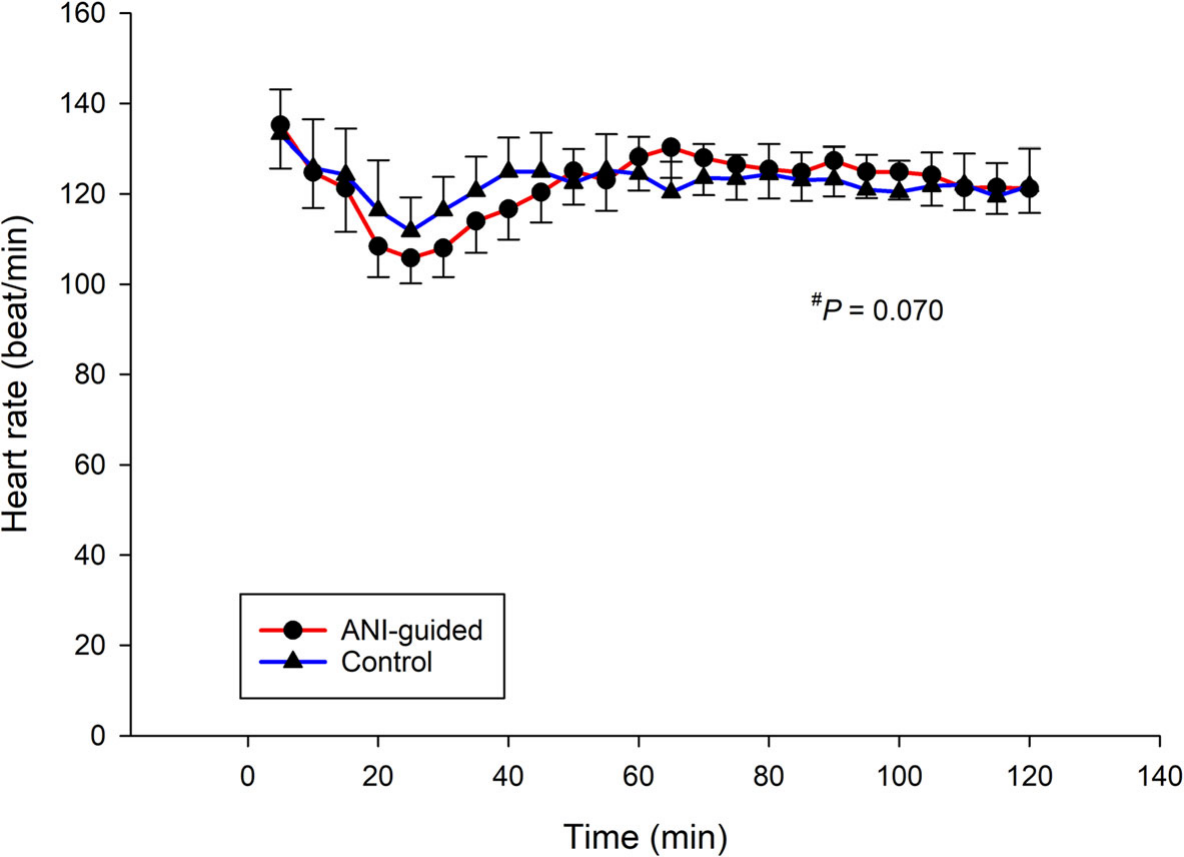
Intraoperative heart rate of both groups. Error bar represents 95% CI. ^#^ linear mixed model. ANI, Analgesia Nociception Index; Time 0, the beginning of surgery

**Fig. 4:**
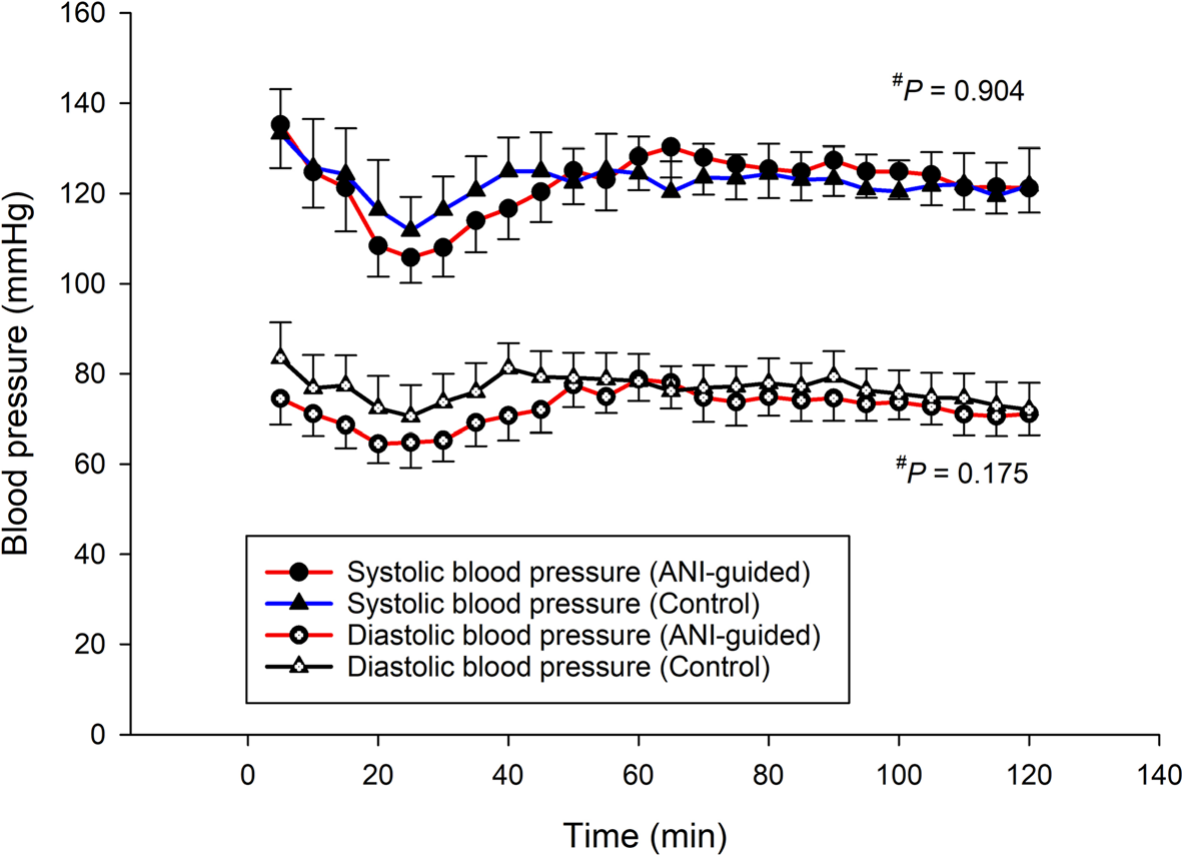
Intraoperative systolic and diastolic blood pressure of both groups. Error bar represents 95% CI. ^#^ linear mixed model. ANI, Analgesia Nociception Index; Time 0, the beginning of surgery

**Fig. 5:**
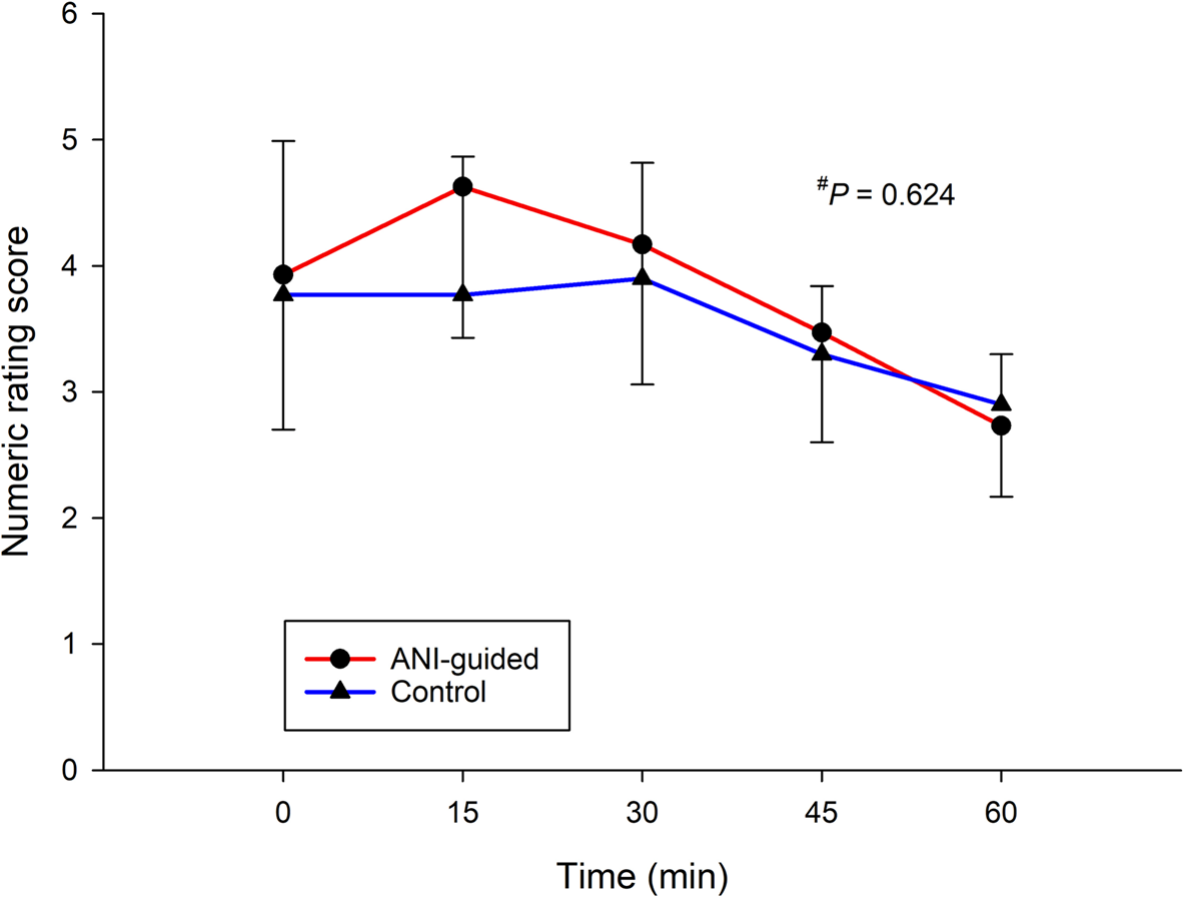
Pain score at PACU during 60 min. Error bar represents 95% CI. ^#^ linear mixed model. ANI, Analgesia Nociception Index; Time 0, immediately admitted in PACU

No difference between groups was evidenced for any of the intraoperative hemodynamic parameters, i.e., heart rate, systolic and diastolic blood pressure (Fig. 3 and Fig. 4). The respective NRS at the PACU among groups was similar (Fig. 5). The respective morphine consumption at the PACU of the ANI-guided and Control groups was not different (5.7 ± 4.9 mg vs 5.5 ± 4.4 mg, *P* = 0.207). The sedation scores at the PACU of both the ANI-guided and Control group were similarly low (0.4 ± 0.5 vs 0.4 ± 0.5, *P* = 0.605). Neither PONV nor postoperative respiratory depression was detected.

## Discussion

The current study reveals that ANI did not decrease intraoperative fentanyl administration in patients undergoing mastectomy. This might be due to the type of surgery chosen. Mastectomy is an intermediate procedure with moderate intraoperative and postoperative acute pain.

The protocol for intraoperative fentanyl administration for the Control group in this study was based on the pharmacokinetic pattern reported by Duthie et al. who showed that a single bolus dose of 100 μg of fentanyl followed by continuous infusion of 100 μg·h^− 1^ resulted in a stable plasma fentanyl concentration of 1–3 ng·mL^− 1^ with effective analgesia [4]. Since mastectomy is an intermediate risk surgery with moderate and constant nociceptive stimulation intensity, we modified the dosage to a lower bolus dose of 75 μg followed by intermittent doses—for practical application—of 25 μg every 30 min with additional doses on demand to avoid over-dosage. We did not include some signs of inadequate analgesia in our protocol (e.g., tears, pupil dilation, and sweat), which may have affected the accuracy of assessment; however, the stable blood pressure and heart rate throughout the intraoperative period of the Control group suggests that the dosage was appropriate. The intraoperative dose of fentanyl guided by ANI was equivalent to the dose administered according to the modified pharmacokinetic pattern. Moreover, the intraoperative ANI scores, immediate postoperative pain scores, and morphine consumption were similar between groups. In Fig. 2, the ANI scores of the ANI-guided group were kept above 50 and maintained at between 50 and 70, while the ANI scores of the Control group were almost parallel and slightly lower. These results confirm that the ANI score can be used as a precise guide for giving a supplement dose of fentanyl and may also serve as a reliable monitor for analgesia intraoperatively. The low pain score and morphine consumption without respiratory depression at the PACU indicates that there was appropriate analgesia in both groups.

Several studies have assessed the ability of ANI to measure nociception or pain. Le Guen et al. reported that the ANI had an inverse linear relationship with visual analogue scores during labor [13]. Ledowski et al. however, found that the ANI did not reflect different levels of postoperative pain in adults undergoing sevoflurane-based anesthesia. These authors explained that the presence of anesthetic drugs and associated sedation of the patient were likely to influence ANI score and proposed that ANI might be more valuable in anesthetized patient [14]. Boselli et al. reported that ANI measured immediately before extubation among adults undergoing general anesthesia using an inhalational agent and remifentanil was significantly associated with pain intensity on arrival in the PACU and the ANI was able to discriminate between patients with a NRS ≤ 3 and NRS > 3 with a high sensitivity (88%) and specificity (83%) [8]. In children under general anesthesia—using desflurane and remifentanil—Sabourdin et al. showed that ANI might provide a more sensitive assessment of nociception than hemodynamic parameters [15]. ANI was demonstrated to be more sensitive than heart rate and systolic blood pressure to reflect nociceptive stimuli in both total intravenous anesthesia and inhalation anesthesia [16, 17]. Recently, Upton et al. conducted a study to compare intraoperative fentanyl guided either by the anesthesiologist’s standard practice or by maintaining an ANI ≥ 50 and reported that the patients receiving ANI-guided intraoperative fentanyl had less pain in PACU, likely as a result of objective fentanyl administration [5].

Unlike the subjective protocol of the control group in the study by Upton et al., the Control group in the current study received intraoperative fentanyl guided by an objective standardized protocol according to the pharmacokinetic pattern of fentanyl [4]. We chose a lower dose regimen of 50 μg·h^− 1^ (i.e., 25 μg every 30 min) with supplemented doses as needed to avoid overdosing, which may result in postoperative sedation and respiratory depression. The pattern of intraoperative heart rate and blood pressure in the Control group were stable throughout the operation (Figs. 3 and 4), indicating that the regimen was appropriate. Similarly, ANI-guided intraoperative fentanyl administration resulted in no differences between groups for any haemodynamic parameter. The heart rate of the ANI-guided group was slightly lower than the Control group with no statistical significance, which may correlate well with the higher mean ANI score in the ANI-guided group. These results indicate that ANI can be used as a proxy of the pharmacokinetic pattern to guide intraoperative fentanyl administration resulting in similar results. The identical low postoperative NRS, sedation, and morphine consumption demonstrate that both regimens provide optimum intraoperative analgesia. Neither PONV or respiratory depression was detected in the current study. The explanation being that the amount of morphine used postoperatively was low and all patients received dexamethasone 8 mg and ondansetron 8 mg to prevent PONV.

Even though we did not use the BIS index, we used a MAC value to monitor and control depth of anesthesia. We adjusted end tidal concentration of desflurane in 50%N_2_O at 3 to 4% to achieve 1 MAC for a given age (MAC_age_) on the monitor [18]. With the additive effect of fentanyl (~ 0.5 MAC) and cisatracurium (~ 0.5 MAC) [19, 20], the total depth of anesthesia was approximately 2 MAC (~MAC-BAR_99_), which is optimal.

The negativity of our results was probably favored by an insufficient statistical power, given that the actual effect size was also smaller than expected. However, we must note that the upper limit of the 95% CI of mean difference was 16.4 μg for the raw values of fentanyl, which is almost half the 25 μg value of the study on which the sample size was based. Since the effect size is very small, there is little chance that increasing the sample size would change the results.

## Limitations

The current study has several limitations. The power calculation of the current study was based on a previous study that had different conditions of anesthesia which might be inappropriate or hazardous. A calculation based on a preliminary study in breast surgery with the similar conditions is thus recommended. We recruited only female patients with an ASA status I-II, the results may not be applied to other groups of patients. The study population included only mastectomy which is an intermediate risk surgery with moderate and constant nociceptive stimulation intensity. Our results may not be generalizable to major surgery with high and fluctuating nociceptive stimulation. Further studies in other groups of patients and different types of surgery are required. We included both simple mastectomy and modified radical mastectomy with lymph node dissection; both of which have different nociception, so choosing one type of surgery might avoid the biases due to type of surgery in a small population.

## Conclusions

Compared with fentanyl administration based on modified pharmacokinetic pattern in patients undergoing mastectomy, ANI did not significantly change intraoperative consumption of fentanyl nor did it change postoperative outcomes. The observation may challenge the use of ANI, unless positive results are evidenced in other surgeries using a more aggressive model.

## Data Availability

The data used to support the findings of this study are available from the corresponding author upon request.

## Abbreviations

ANI: Analgesia Nociception Index
CI: Confidence interval
PACU: Post-anesthetic care unit
BIS: Bispectral index
MAC: Minimum alveolar concentration
TOF: Train-of-four
PONV: Postoperative pain, nausea and vomiting
ECG: Electrocardiogram
HRV: Heart rate variability
RSA: Respiratory sinus arrhythmia
CONSORT: Consolidated Standards of Reporting Trials
NSAID: Nonsteroidal anti-inflammatory drug
N_2_O: Nitrous oxide
O_2_: Oxygen
NRS: Numeric rating scale
SD: Standard deviation
ANOVA: Repeated measures analysis of variance
BMI: Body mass index
ASA: American Society of Anesthesiologists
SBP: Systolic blood pressure
HR: Heart rate

## References

[1] Oliveira CR, Bernardo WM, Nunes VM (2017). Benefit of general anesthesia monitored by bispectral index compared with monitoring guided only by clinical parameters. Systematic review and meta-analysis. Braz J Anesthesiol.

[2] Aranake A, Mashour GA, Avidan MS (2013). Minimum alveolar concentration: ongoing relevance and clinical utility. Anaesthesia..

[3] Murphy GS (2018). Neuromuscular monitoring in the perioperative period. Anesth Analg.

[4] Duthie DJ, McLaren AD, Nimmo WS (1986). Pharmacokinetics of fentanyl during constant rate i.v. infusion for the relief of pain after surgery. Br J Anaesth.

[5] Upton HD, Ludbrook GL, Wing A, Sleigh JW (2017). Intraoperative "analgesia nociception index"-guided fentanyl administration during Sevoflurane anesthesia in lumbar discectomy and laminectomy: a randomized clinical trial. Anesth Analg.

[6] Boselli E, Jeanne M (2014). Analgesia/nociception index for the assessment of acute postoperative pain. Br J Anaesth.

[7] Abad-Gurumeta A, Ripolles-Melchor J, Casans-Frances R, Calvo-Vecino JM (2017). Monitoring of nociception, reality or fiction?. Rev Esp Anestesiol Reanim.

[8] Boselli E, Bouvet L, Begou G, Dabouz R, Davidson J, Deloste JY (2014). Prediction of immediate postoperative pain using the analgesia/nociception index: a prospective observational study. Br J Anaesth.

[9] Daccache G, Caspersen E, Pegoix M, Monthe-Sagan K, Berger L, Fletcher D (2017). A targeted remifentanil administration protocol based on the analgesia nociception index during vascular surgery. Anaesth Crit Care Pain Med.

[10] Dundar N, Kus A, Gurkan Y, Toker K, Solak M (2018). Analgesia nociception index (ani) monitoring in patients with thoracic paravertebral block: a randomized controlled study. J Clin Monit Comput.

[11] Turan G, Ar AY, Kuplay YY, Demiroluk O, Gazi M, Akgun N (2017). Analgesia nociception index for perioperative analgesia monitoring in spinal surgery. Rev Bras Anestesiol.

[12] Ali Hassn AM, Zanfaly HE, Biomy TA (2016). Pre-emptive analgesia of ultrasound-guided pectoral nerve block II with dexmedetomidine–bupivacaine for controlling chronic pain after modified radical mastectomy. Res Opin Anesth Intensive Care.

[13] Le Guen M, Jeanne M, Sievert K, Al Moubarik M, Chazot T, Laloe PA (2012). The analgesia nociception index: a pilot study to evaluation of a new pain parameter during labor. Int J Obstet Anesth.

[14] Ledowski T, Tiong WS, Lee C, Wong B, Fiori T, Parker N (2013). Analgesia nociception index: evaluation as a new parameter for acute postoperative pain. Br J Anaesth.

[15] Sabourdin N, Arnaout M, Louvet N, Guye ML, Piana F, Constant I (2013). Pain monitoring in anesthetized children: first assessment of skin conductance and analgesia-nociception index at different infusion rates of remifentanil. Paediatr Anaesth.

[16] Jeanne M, Clement C, De Jonckheere J, Logier R, Tavernier B (2012). Variations of the analgesia nociception index during general anaesthesia for laparoscopic abdominal surgery. J Clin Monit Comput.

[17] Ledowski T, Averhoff L, Tiong WS, Lee C (2014). Analgesia nociception index (ANI) to predict intraoperative haemodynamic changes: results of a pilot investigation. Acta Anaesthesiol Scand.

[18] Nickalls RW, Mapleson WW (2003). Age-related iso-MAC charts for isoflurane, sevoflurane and desflurane in man. Br J Anaesth.

[19] Vereecke HE, Proost JH, Heyse B, Eleveld DJ, Katoh T, Luginbuhl M (2013). Interaction between nitrous oxide, sevoflurane, and opioids: a response surface approach. Anesthesiology..

[20] Sebel PS, Glass PS, Fletcher JE, Murphy MR, Gallagher C, Quill T (1992). Reduction of the MAC of desflurane with fentanyl. Anesthesiology..

